# Lithium Metal Battery Quality Control via Transformer–CNN Segmentation

**DOI:** 10.3390/jimaging9060111

**Published:** 2023-05-31

**Authors:** Jerome Quenum, Iryna V. Zenyuk, Daniela Ushizima

**Affiliations:** 1 Department of Electrical Engineering and Computer Science, Berkeley College of Engineering, University of California, Berkeley, CA 94720, USA; 2 Applied Mathematics and Computational Research Division, Lawrence Berkeley National Laboratory, Berkeley, CA 94720, USA; 3 Department of Chemical & Biomolecular Engineering, National Fuel Cell Research Center, University of California Irvine, Irvine, CA 92697, USA; 4 Berkeley Institute for Data Science, University of California Berkeley, Berkeley, CA 94720, USA; 5 Bakar Computational Health Sciences Institute, University of California San Francisco, San Francisco, CA 94143, USA

**Keywords:** deep learning, semantic segmentation, quality control, Transformer–CNN, battery

## Abstract

Lithium metal battery (LMB) has the potential to be the next-generation battery system because of its high theoretical energy density. However, defects known as dendrites are formed by heterogeneous lithium (Li) plating, which hinders the development and utilization of LMBs. Non-destructive techniques to observe the dendrite morphology often use X-ray computed tomography (XCT) to provide cross-sectional views. To retrieve three-dimensional structures inside a battery, image segmentation becomes essential to quantitatively analyze XCT images. This work proposes a new semantic segmentation approach using a transformer-based neural network called TransforCNN that is capable of segmenting out dendrites from XCT data. In addition, we compare the performance of the proposed TransforCNN with three other algorithms, U-Net, Y-Net, and E-Net, consisting of an ensemble network model for XCT analysis. Our results show the advantages of using TransforCNN when evaluating over-segmentation metrics, such as mean intersection over union (mIoU) and mean Dice similarity coefficient (mDSC), as well as through several qualitatively comparative visualizations.

## 1. Introduction

Lithium metal batteries (LMBs) offer high specific energy density as Li is a light element. Furthermore, the liquid electrolyte is not needed in LMBs and polymer or ceramic electrolytes can be used, which are inherently more safe compared to organic electrolytes. However, currently, no commercially available LMB systems exist because of Li dendrite formation resulting from inhomogeneous Li metal plating. This section describes the current methods for imaging batteries, followed by a review of the main algorithms for image analysis, semantic segmentation, and battery characterization.

### 1.1. Assessing Battery Quality with Imaging

Synchrotron-based hard X-ray computed tomography (XCT) has spatial resolution suitable to resolve dendrite structures that have microscale dimensions [1,2]. Lithium has a low atomic number and, therefore, a low X-ray attenuation. For example, Li dendrites will appear to be void spaces within dense polymer electrolyte materials. XCT imaging of LMB seldom recovers chemical information and relies on differences in material thicknesses and atomic numbers to differentiate Li from solid polymer electrolytes (SPE). To worsen the detection of LMB dendrites, they are porous formations that lead to large intensity variations within their volume. When the LMB is subjected to multiple charge–discharge cycles, a phenomenon known as pitting corrosion develops, and an electrode can present a combination of pits and dendrites, with both presenting similar X-ray attenuation in XCT data. Thus, it is challenging to differentiate dendrites from pits but also to quantify them properly.

Previous LMB *operando* studies using XCT analyzed Li metal plating and how the interphase evolves in symmetric Li–Li cells with polymer electrolytes and in the batteries with Li metal anode [2,3,4,5,6]. These studies focused on the battery design and functioning; however, they lack methods for revealing the structure of the dendrites. Most of these previous studies applied traditional thresholding algorithms to conduct segmentation, which is, unfortunately, not reproducible when applied to new samples and rarely applicable to the differentiation between Li metal, pits, and other materials. Alternatively, manual segmentation could be used for a selected cross-section, but it is often unfeasible for full-stack high-resolution imaging surveillance because it is a highly time- and labor-intensive process [7].

### 1.2. Deep Learning for Semantic Segmentation

Semantic segmentation is the computer vision task of splitting an image into different categories [8] using a data-driven model to assign a pixel-wise classification given the input image. Different models have been proposed, among them being fully convolutional networks (FCN) [9], which proposed a paradigm shift in 2015, running fully connected layers, and includes a way to allow for each pixel to be classified from feature maps coming from convolutional layers. This builds on a series of local convolutions of preceding layers that aim to obtain a representation of multi-scale feature maps used for the classification tasks. Around the same time, Ronneberger et al. [10] introduced their convolutional neural network (CNN), a new CNN-based encoder–decoder model known as U-Net, and showed that combining higher and lower features symmetrically is beneficial in obtaining better performance. Soon after, SegNet [11] and Deeplab [12] were proposed, confirming that the encoder–decoder architecture is well suited for such a task.

Many of these works [12,13,14,15] also leverage the *atrous convolution* to show that it could help to capture contextual information. In particular, Y-Net [15] used three modules to improve segmentation accuracy. In addition to the Regular Convolution Module and the Pyramid Pooling Module, which allows the model to learn global information about potential locations of the target at different scales, the Dilated Convolution Module took advantage of the fact that the target is often shared out in the samples, which supports learning sparse features in their structure. PSPNet [14], on the other hand, adds ResNet [16] as a backbone, while multi-scale feature maps are aggregated in its encoder.

Although these architectures work well, the computer vision community has increasingly witnessed their design shift from pure CNN-based design with [9,10,11,12,14,15] to transformer-based designs, which started with ViT [17,18,19,20,21]. Later on, hybrid models started exploiting the best of both worlds by either using a transformer as an encoder and CNN as a decoder [22,23], or CNNs as encoders and a transformer as a decoder, or even using CNNs and encoder–decoder while transformers are used in the middle or in between to process the feature maps. One such hybrid model is HRNet-OCR [24], which has a CNN as a backbone and combines it with cross-attention layers between features of different scales to account for multiple contexts and scales in the data.

In all these schemes, the common denominator remains the attention mechanism, which has proven in the past few years to exceed the performance of models disregarding it. That is because it allows the features not to be subject to the inductive biases and translation invariance that occur in CNNs. Instead, it allows the model to learn long-term dependencies between pixel locations [17]. In other words, it allows for a better representation by leveraging contextual information either between pixels, patches, or channels.

### 1.3. Problem and Motivation

**Figure 1:**
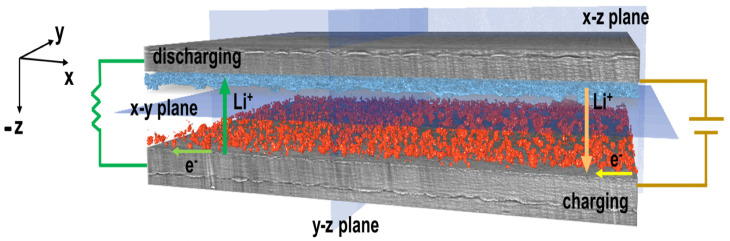
Diagram illustrating the Li–polymer–Li symmetric cell design, imaged using X-ray CT, with highlighted dendrite formations (blue) and the redeposited Li (red).

Li dendrite formations initiate during battery cycling, as illustrated in Figure 1, with dendrites nucleating on the interface between the electrolyte and the electrodes. Dendrite growth depends on the current density of plating, electrolyte transference number, electrolyte mechanical properties, and impurities present in Li metal material and at the interfaces. Earlier works have shown that increasing the shear modulus of the SPEs can help to suppress Li dendrite growth but cannot fully eliminate it [25,26,27,28]. Further studies have shown that Li metal surface impurities (Li${}_{3}$N, Li${}_{2}$CO${}_{3}$, and Li${}_{2}$O) can result in inhomogeneous current density and promote nucleation of Li dendrites.

Accurate segmentation for measuring dendrite volume has guided research and quality control of battery designs, as well as tests of materials used for its components. Deep learning methods can provide exceptional segmentation results [29,30,31] when using high-resolution XCT data, particularly when large collections of annotated data are available. For example, Kodama et al. [32] and Muller et al. [33] proposed U-Net-based architectures for segmentation of lithium ion batteries (LIB), being the first article focused on segmenting samples into three phases: nickel manganese cobalt oxide, solid electrolyte, and void, and the second article described segmentation of graphite–silicon composite electrodes. More recent work by Zhang et al. [34] used a CNN known as D-LinkNet to inspect the effect of distortion on the segmentation accuracy of LIB samples. Additional works by the same team [35] used a U-Net for multiphase segmentation of battery electrodes from nano-CT images. Despite being focused on battery segmentation, those studies lack information on dendrite segmentation.

Previous studies [36,37] on inspecting dendrites in batteries discussed problems regarding the mechanisms and types of nucleation, e.g., lateral growth or Li filaments. Data acquisition modes range from electron microscopy [36,38,39] to XCT [37,39] with valuable morphological characterization and designs for suppression of dendrite growth, but dendrite detection was addressed mostly qualitatively through dendrite projections and/or visualizations. For example, the dendrite volume calculation in [39] was based on median filter and Otsu thresholding, a method that seldom works for more than a few slices from an XCT stack, unless considering strenuous manual postprocessing [40].

### 1.4. Research Contributions

**Figure 2:**
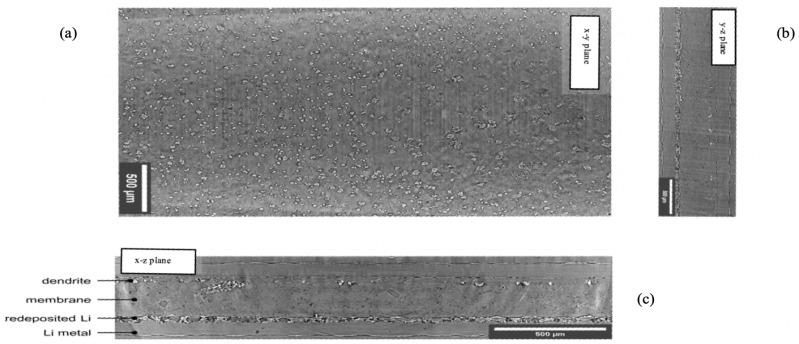
Cross- sectional images for the Li–polymer–Li symmetric cell; (**a**) cross section of the x–y plane where the training was completed on this plane; (**b**) cross sections of the x–z plane and detailing of the cell components; (**c**) cross sections of the y–z plane.

The proposed research describes the design and implementation of dendrite and Li deposit segmentation from 3D XCT images and the contribution is two-fold: this work uses LMB, a more modern battery design in comparison with LIB, and the proposed semantic segmentation model, which builds upon a hybrid Transformer–CNN architecture never used before in LMB analysis. In performing the segmentation of a 3D volume automatically, we could either design a 3D model that performs directly on an input volume or we could leverage 2D models by subdividing the volume into slices. This paper introduces a 2D model due to its ability to be trained faster and with a limited number of samples, hence making it more versatile. In particular, we propose an architecture that benefits from both the contextual information learned from transformers and the global information captured by CNNs to predict dendrites and Li deposits (Figure 2) from a lithium metal battery that underwent cycling and was imaged using high-resolution XCT data. This article compares four different deep learning architectures, including U-Net, Y-Net, TransforCNN, and E-Net, on their ability to segment dendrites inside the cycled symmetric Li–Li battery with polymer electrolytes.

## 2. Materials and Methods

Li metal holds a high theoretical capacity (3860 mAh/g) and a large negative thermodynamic potential (−3.06 V vs. SHE) [41]. Thus, it is considered a promising candidate for the next-generation battery anode. Li metal is highly active and can introduce a series of side reactions in a battery system with liquid electrolytes. This can also cause the dendrite to form, which would eventually lead to short circuits and introduce safety issues to the battery. Using solid electrolytes instead of liquid electrolytes, a more stable interface can be designed between the electrolyte and the Li metal electrodes, thus alleviating the dendrite formation issue. Recent developments in electrolyte engineering can be found in [35,39,42,43].

Currently, several solid-state materials are used as electrolytes and separators, such as polymers and single-ion conducting inorganic solid electrolytes (glass or ceramic). Polymer materials are promising as they are mechanically flexible because polymer materials can be produced in a roll-to-roll scalable process and be designed very thinly. However, for SPEs to have broad deployment, strategies for dendrite suppression must be developed, such as coatings and soft interlayers, including polymers as well as ionic liquids [44,45,46].

Design of interfaces in Li metal batteries or all solid-state batteries (ASSBs) is challenging as it involves control of Li ion plating onto Li metal and this plating process needs to be uniform. When not completed properly, interactions at the interfaces of electrodes arise due to transport processes associated with ions [43,47], which can form ionic aggregates. Dendrite growth can be triggered by the formation of localized regions of high lithium ion concentration, which can occur due to the clustering of lithium ions into ionic aggregates. In general, dendrites are formed during battery charge and discharge cycles. This happens especially when a battery is charged at high current densities due to the heterogeneous Li metal plating even when considering solid electrolytes. Tracking the structure and evolution of dendrites is important to develop a strategy to prevent their growth. Dendrites are tree-like and porous structures, usually with a size in nano to micro scale. Given the morphological structure of dendrites and their size relative to an input stack, performing XCT segmentation is a suitable method of analysis as it allows for a pixel-wise classification, which helps to quantify the volume of dendrites and use it as a proxy of battery quality.

### 2.1. Electrochemical Testing

Li/Li symmetric cells are assembled using two Li metal electrodes and are considered a tool for testing and observing the Li metal anode without being affected by cathode materials.

**Figure 3:**
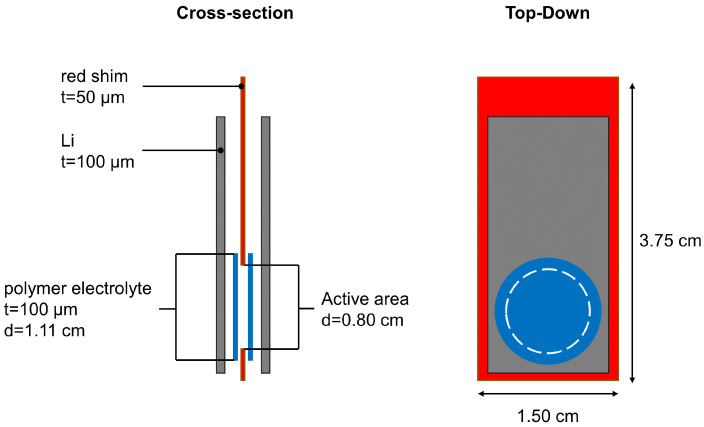
Schematic illustration of the pouch cell.

Free-standing Li metal foils with a thickness of 100 μm from FMC/Livent™ were used. Polymer electrolyte membrane was sourced from an industrial partner with a thickness of 140 μm as a research sample. The cell was assembled as Figure 3 shows. A red shim with a thickness of 50 μm was used to create a circle with 0.8 cm diameter. Two circular polymer electrolytes with a diameter of 0.80 cm were punched out and placed on each side of the red shim. Two Li metal foils were then placed on the outside of the membranes as electrodes. The electrodes were connected to the metal tabs. The cell was sealed with a vacuum sealer. A current density of 1.5 mA/cm${}^{2}$ was periodically applied to the cell for 10 min, and the battery rested for 20 min. The cell was cycled at 3.0 mAh/cm${}^{2}$ for one full cycle (120 min for charging and 120 min for discharging), after which the cell XCT scan was acquired.

### 2.2. Synchrotron X-ray CT Imaging

The XCT scan was acquired at Beamline 2-BM at Advanced Photon Source (APS) at Argonne National Laboratory (ANL), which used a 20 μm LuAG scintillator with 5× lenses and an sCMOS PCO edge camera. A 27.5 keV energy was selected using a multilayer monochromator, with 100 ms exposure time per back projection and over 180 degrees of rotation, enabling the collection of 1500 projections. The pixel size is 1.33 μm and the field of view is 3.3 mm. Three FOVs were recorded and were stitched together to form a vertical height of >3 mm during the postprocessing. Tomographic reconstructions considered TomoPy version 1.14 with Gridrec algorithm [48,49,50].

### 2.3. Raw Data Preprocessing

**Figure 4:**
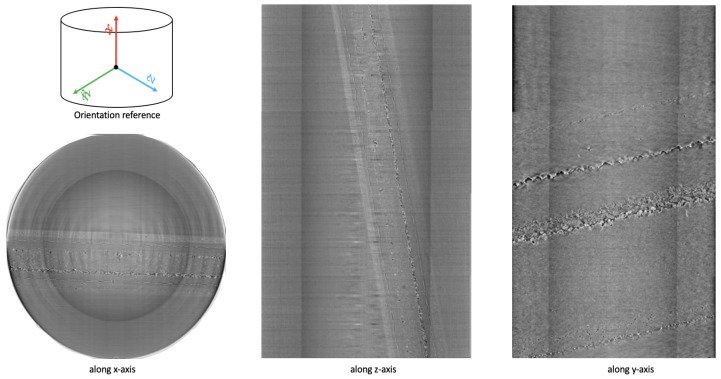
Sample raw data obtained after TomoPy reconstruction of a CT scan.

The resulting raw TomoPy reconstruction was a large volume of size (3977,2575,2582) and required additional alignments to correct for the various motion involved in collecting the data, as shown in Figure 4. As these raw data contain a great deal of noise and irrelevant parts, we proceed to develop an algorithm that will allow us to cleanly crop out those regions. In the process, we inverted the grayscale volume and obtained a maximum projection [40] image on the stacks to facilitate our ability to locate corners.

**Figure 5:**
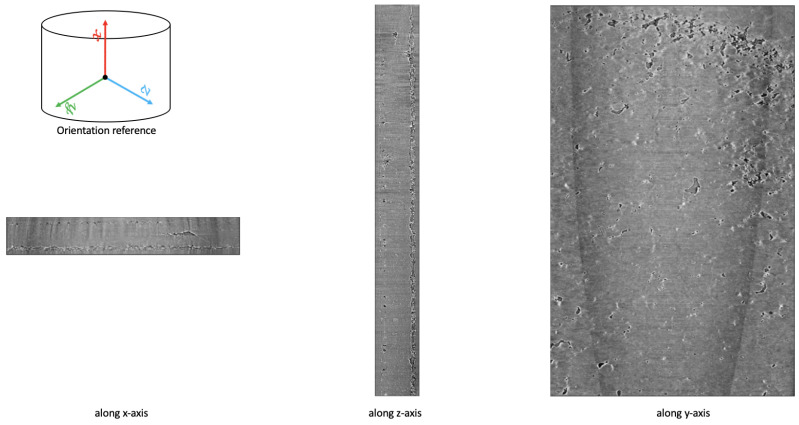
Sample region of interest (RoI) data obtained after preprocessing TomoPy reconstruction of a CT scan.

To align the data, we first used a series of perspective transformations and homography to rectify the region of interest along each plane. Although this process could be automated using feature detectors and feature matching techniques, such as MOPS [51] and SIFT [52], we manually selected corners for optimal precision. We then rectified and cropped the raw data to obtain the region of interest to a volume of size (3849,340,2071), as shown in Figure 5.

### 2.4. Deep Learning: CNN and Vision Transformer

**Figure 6:**
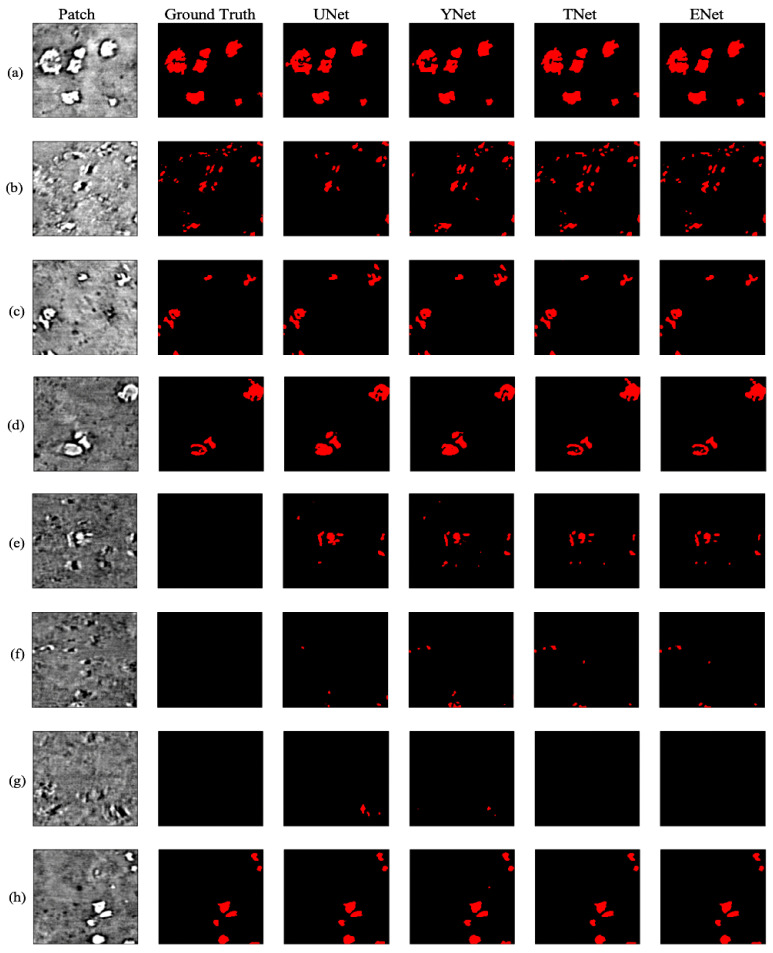
XCT cross–sections and corresponding segmentation results for U-Net, Y-Net, T-Net (TransforCNN) with inputs in 1st column characterized by: (**a**) high concentration of large Li agglomerates, (**b**) moderate concentration of small Li agglomerates, (**c**) low concentration of small Li agglomerates, (**d**) low concentration of large Li agglomerates, (**e**–**g**) low concentration of small Li agglomerates that were not labeled by humans but detected by the deep learning algorithms, (**h**) low concentration of small Li agglomerates.

For a given volume stack, we apply 2D models due to their versatility. In doing so, we subdivide each training hand-labeled slice into 128×128 patches, which were then separated into training, validation, and testing datasets. Based on their known performances over the years, we investigate CNN-based architectures, such as U-Net [10] and Y-Net [15]. In addition, we also compared their performance with TransforCNN, our proposed transformer encoder-based network, and E-Net, an ensemble network over U-Net, Y-Net, and TransforCNN. We train the networks in a weakly supervised fashion where a small subset of labeled data was used in conjunction with a much larger unlabeled sample size. Figure 6 shows a sample output of the model considered in this work.

#### 2.4.1. U-Net

**Figure 7:**
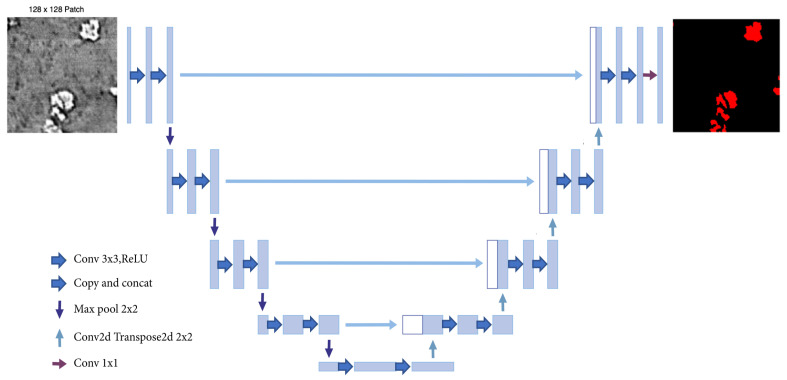
U-Net architecture.

Figure 7 shows a U-Net, a CNN architecture that was first introduced in 2015 by Ronneberger et al. for the semantic segmentation of biomedical images. The original work [10] proposes an architecture that consists of a contracting path to capture context and a symmetric expanding path that enables precise localization. In our work, the model was adapted to take images of size 128×128 as input. For the encoder, we started with 16-channel 3×3 kernels and double the number at each layer, followed by a ReLU activation and max pooling until a 256-channel 8×8 resolution feature map is obtained. We reversed the operation with transposed convolutions operation on the decoder side and concatenate with corresponding size encoder feature maps until the desired output shape is obtained.

#### 2.4.2. Y-Net

**Figure 8:**
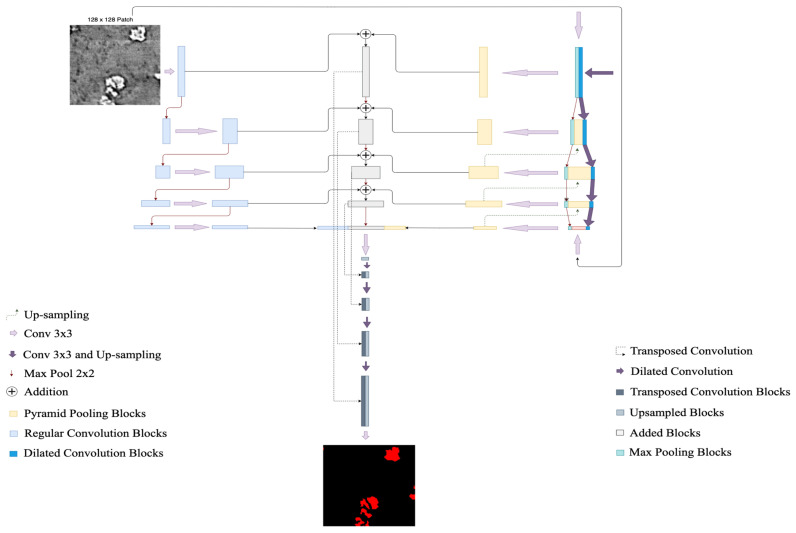
Y-Net architecture.

Figure 8 shows Y-Net, a CNN architecture originally introduced by Quenum et al. [15] to segment barcodes from ultra-high-resolution images. By leveraging Y-Net’s architecture, we have modified and adapted the Regular Convolution Module to take in 128×128 images from training slices. As it consists of convolutional and pooling layers, we started with 24-channel 3×3 kernels and doubled the number at each layer. We alternated between convolution and max pooling until we reached a feature map size of 8×8 pixels. The Dilated Convolution Module here took advantage of the fact that dendrites are often shared out in the samples to learn sparse features in their structure. It also took 128×128 input patches and we maintained 16-channel 3×3 kernels throughout the module while the dimensions of the layers were gradually reduced using a stride of 2 until a feature map of 8×8 pixels is obtained. Finally, the Pyramid Pooling Module, which allows the model to learn global information about potential locations of the dendrites at different scales, had its layers concatenated with the layers on the dilated convolution module to preserve the features extracted from both modules.

#### 2.4.3. TransforCNN

**Figure 9:**
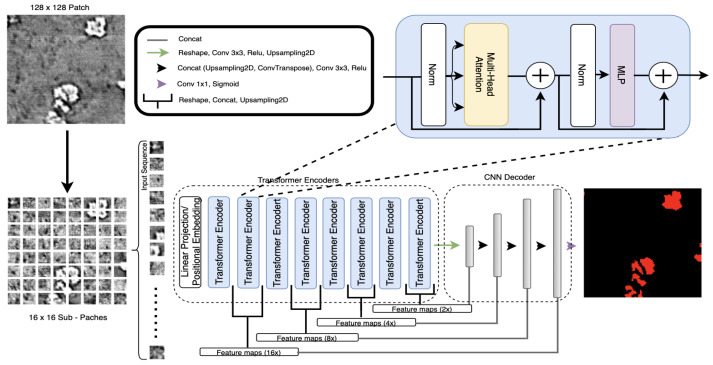
TransforCNN architecture combines the encoder of Vision Transformers with the CNN decoder.

TransforCNN is a hybrid Transformer-CNN segmentation model that leverages the encoder model of Vision Transformers ViT [17] and the decoder architecture of CNNs. More specifically, its encoder model was first introduced in natural language processing (NLP) by Vaswani et al. [53] and its multi-headed self-attention was later shown (by ViT) to help remove the common inductive biases observed in CNN-only models by relating all input sequences with each other. As depicted in Figure 9, the proposed architecture is hybrid because it combines the Transformer Encoders Block with the CNN Decoder Block to deliver semantic segmentation.

The Transformer Encoders Block takes inputs that are 16×16 sub-patch sequences from the 128×128 patches that were obtained from training slices. These patches are flattened and each is embedded into a 64-dimensional feature vector via a linear projection and is added to its corresponding Fourier features (FF) positional encoding. We used 8 transformer encoder units and the outputs of every 2 transformer encoders were reshaped into a 2-dimensional feature map representation, concatenated, up-sampled, and recombined with the layers from the CNN Decoder Block of corresponding dimension.

The CNN Decoder Block takes in the output of the last transformer encoder unit and reshapes it into a 2-dimension representation on which a set of 3×3 kernels convolutions and max pooling is applied to obtain a feature map of 8×8 pixels. The resulting feature maps are then concatenated with corresponding size feature maps coming from the Transformer Encoder Block and up-sampled continuously until the final output is obtained. This last step allows for the enhancement of the features in the CNN Decoder Block as we are progressively reconstructing the output dimension of 128×128.

#### 2.4.4. E-Net

We have combined the results of different architectures, namely U-Net, Y-Net, and TransforCNN, to create an ensemble prediction scheme called E-Net. It was found that our best mean intersection over union (mIoU) is obtained when combining 20% of U-Net with 80% of TransforCNN, while the best mean Dice similarity coefficient (mDSC) is obtained using only a TransforCNN. This was achieved by weighing the predicted segments of each of the models with coefficients in the interval [0,1] in increments of 0.1 and evaluating all possible combinations against the available ground truth segments.

## 3. Results

In training the models (U-Net, Y-Net, and TransforCNN), we used one NVIDIA Tesla V100 GPU for each experiment. We obtained a total of 4433 samples of resolution 128×128 with their corresponding hand-labeled ground truth that the models were trained on. We used 80% of the examples for the training set, 10% for the validation set, and 10% for the testing set. We used data augmentation schemes in training all models, which consist of random rotations in all directions, random flips (vertically and horizontally), random cropping (2%), random shifts, random zoom (range in [0.8, 1]), and a small range of random brightness and contrast variation (+/− 5%). We trained the U-Net for 450 epochs, while the Y-Net and TransforCNN models were trained for 130 and 300 epochs, respectively. The training process took an average of 1.5 GPU days for U-Net because it has slightly fewer parameters than Y-NET, which took about 2 GPU days. TransforCNN has significantly more parameters (∼3 × the size of U-Net) and it took about 3 GPU days to converge. We used the Adam optimizer for U-Net and Y-Net and used the AdamW optimizer for TransforCNN; AdamW is a stochastic optimization method that modifies the standard implementation of weight decay in Adam by decoupling weight decay from the gradient update.

**Figure 10:**
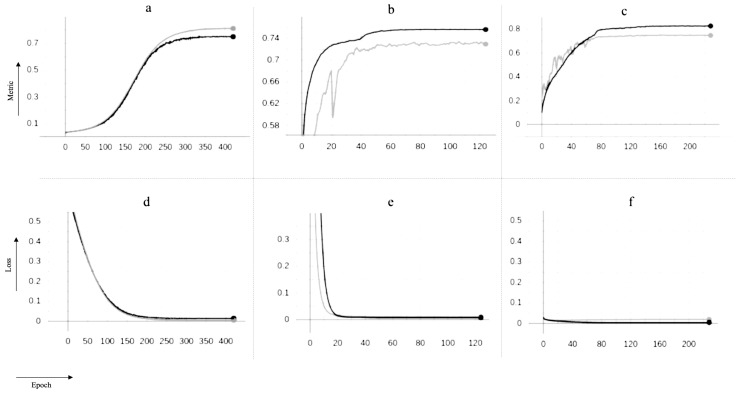
Training curves for U-Net, Y-Net, and TransforCNN on the *y*-axis vs. numbers of epochs on the *x*-axis; the models were optimized over the binary cross-entropy function as loss and evaluated on the Dice similarity coefficient as evaluation metric during training. The gray curves depict behavior on the validation sets while the black curves show behavior on the training sets over increasing numbers of epochs; (**a**) training and validation Dice coefficient for U-Net; (**b**) training and validation dice coefficient for Y-Net; (**c**) training and validation Dice coefficient for TransforCNN; (**d**) training and validation loss for U-Net; (**e**) training and validation loss for Y-Net; (**f**) training and validation loss for U-Net; the use of dropout during only the training phase explains why the models tend to perform better on the validation set over time.

As shown in Figure 10, Y-Net and TransforCNN converge faster than U-Net, with the initial loss of the TransforCNN model being significantly lower than that of the U-Net and Y-Net models. We have experimented with various loss functions, such as Tversky loss [54] described in Equation (1), the focal Tversky loss [55] described in Equation (2), the binary cross-entropy loss (Equation (3)), and the balanced cross-entropy loss described in Equation (4), out of which the binary cross-entropy loss yields the best results. One interesting observation is that, although the validation curve on U-Net exhibits characteristics of better generalization, the quantitative results show otherwise.

For evaluation, we have used the Dice similarity coefficient described in Equation (5) and the Jaccard index, also known as intersection over union (Equation (6)). In all the equations, *y* and $\hat{y}$ are, respectively, the ground truth and prediction on patch *i*, and TP, FP, and FN represent the number of true positives, false positives, and false negatives, respectively. Note that Equation (6) could also be expressed as *DSC*/(2-*DSC*).

(1) $$\begin{array}{c} L_{\mathrm{Tversky}}\left(y,\hat{y}\right)=\frac{y\hat{y}}{y\hat{y}+\beta \left(1-y\right)\hat{y}+\left(1-\beta \right)y\left(1-\hat{y}\right)} \end{array}$$

where α,β>0,α+β=1.

(2) $$\begin{array}{c} L_{\mathrm{Focal}\;\mathrm{Tversky}}\left(y,\hat{y}\right)={\left[1-L_{\mathrm{Tversky}}\left(y,\hat{y}\right)\right]}^{\gamma } \end{array}$$

where α,β>0,α+β=1,γ=4/3.

(3) $$\begin{array}{c} L_{\mathrm{Cross}\;\mathrm{Entropy}}\left(y,\hat{y}\right)=-y\log\left(\hat{y}\right)-\left(1-y\right)log\left(1-\hat{y}\right) \end{array}$$

(4) $$\begin{array}{c} L_{\mathrm{balanced}\;\mathrm{Cross}\;\mathrm{Entropy}}\left(y,\hat{y}\right)=-\beta y\log\left(\hat{y}\right)-\left(1-\beta \right)\left(1-y\right)log\left(1-\hat{y}\right) \end{array}$$

where β∈[0,1].

(5) $$\begin{array}{c} \mathrm{DSC}\left(y,\hat{y}\right)=\frac{2TP}{2TP+FP+FN} \end{array}$$

(6) $$\begin{array}{c} IoU\left(y,\hat{y}\right)=\frac{TP}{TP+FP+FN} \end{array}$$

## 4. Discussion

To assess the performance of all the semantic segmentation models, we use the mean Dice similarity coefficient (mDSC) and the mean intersection over union (mIoU) as metrics, as shown in Table 1. As indicated, our proposed pipeline outperforms U-Net [10] and Y-Net [15] by a mIoU of 8.13% and 10.3% and mDSC of 6.49% and 8.57%, respectively. Shown as well in Table 1 is a slight mIoU improvement of 0.03% by our ensemble network analysis (E-Net) on TransforCNN.

In addition, Table 1 displays that, while TransforCNN is successful in segmenting out dendrites, its latency is at least 3.16× slower than U-Net, which has the fastest latency of all models evaluated at 65.36 milliseconds (ms). The slowest of all the models is observed to be E-Net, which performs 7.24× slower than U-Net.

**Figure 11:**
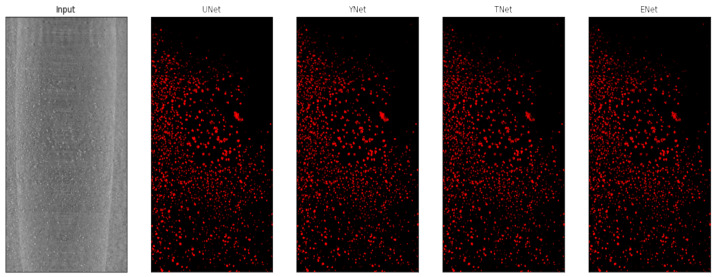
XCT cross-section along the x–y plane and corresponding segmentation results for U-Net, Y-Net, T-Net (TransforCNN), and E-Net.

**Figure 12:**
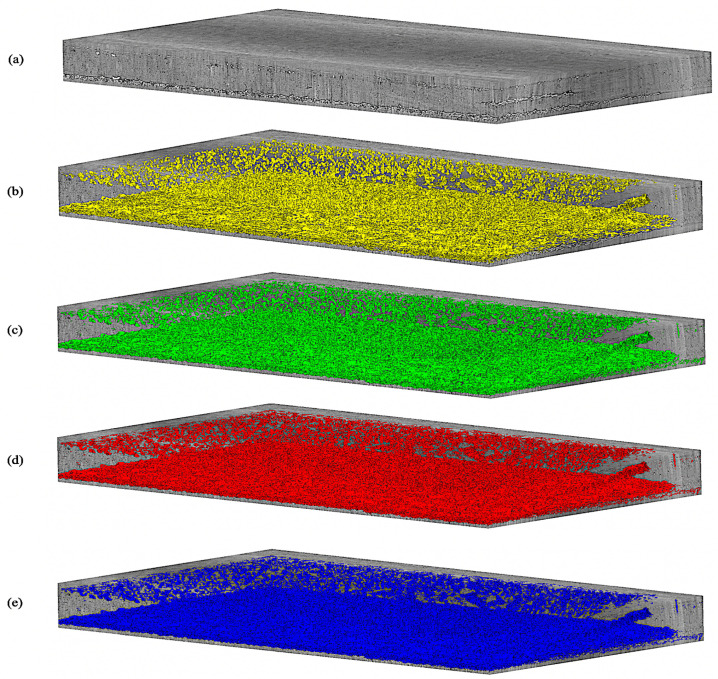
3D rendering of U-Net, Y-Net, T-Net (TransforCNN), and E-Net on test volume; (**a**) grayscale test input volume; (**b**–**e**) are, respectively, U-Net, Y-Net, T-Net (TransforCNN), and E-Net predictions.

Qualitatively, Figure 11 shows the predictions on an unseen test slice, while Figure 6 shows sample predictions at the patch level from previously labeled images. As observed in the first and second rows (a; b), the TransforCNN and U-Net predictions are the closest to the ground truth. The third, fourth, and eighth rows (c; d; h) show that U-Net and Y-Net tend to generalize better as the unlabeled dendrite regions in the input patches are segmented out by these two models, while TransforCNN and E-Net still reflect the ground truth images. The fifth, sixth, and seventh rows (e; f; g) show the generalization potential of all the models, while the predictions of TransforCNN and E-Net overall tend to remain closer to the ground truth. In addition, Table 2 summarizes the absolute number of voxels corresponding to the segmented dendrite and redeposited Li volume as well as their volume fraction and Figure 12 shows the 3D rendering of stack using all methods.

## 5. Conclusions

LMBs are promising candidates for next-generation batteries because of their high specific energy density. Currently, Li dendrites growth is an issue as it can lead to loss of Li (dead Li), shorting of cells, and other undesirable degradation phenomena.

More specifically, short-circuiting is a leading failure mechanism in LMBs due to the uncontrolled propagation of lithium protrusions that often present a dendritic morphology. The energy density benefits of using LMBs can only be harvested after scientists are able to detect and regulate the dendrite formation and control dendrite growth.

Uneven lithium ion distribution and dendrite formation can compromise battery performance and safety. For this reason, this paper introduced a semantic segmentation algorithm called TransforCNN that detects both dendrites and re-deposited Li accurately and compared it with traditional approaches.

Overall, it was observed that TransforCNN and E-Net tend to learn semantics in the ground truth images provided during training. In contrast, U-Net and Y-Net tend to simply generalize even to cases where segments in the ground truth were wrongly hand-labeled. We speculate that this may lead to U-Net and Y-Net being wrongly penalized during the evaluation process while TransforCNN and E-Net are rewarded since their predictions always appear to be the closest to the ground truth.

Experiments have also illustrated that our approach outperforms the existing methods, although it is slower than the fastest (U-Net) of all the considered models. In future work, we aim to extend this method to a multi-class segmentation task, differentiating dendrites from pits and other lithium deposits while improving the current latency in a weakly supervised fashion.

## Figures and Tables

**Table 1:** mIoU, mDSC, inference time, and patch size for Li–Li symmetric battery dataset; bold numbers emphasize best performance.

	mIoU	mDSC	Latency (ms)	Input Patch Dimensions (px)
U-Net	0.8698	0.8998	**65.36**	128×128
Y-Net	0.8481	0.8790	103.62	128×128
TransforCNN	0.9511	**0.9647**	206.75	128×128
E-Net	**0.9514**	0.9641	473.59	128×128

**Table 2:** Volume and percentage volume occupied for U-Net, Y-Net, TransforCNN, and E-Net.

	Volume (Cubic Px)	Occupied Volume Percentage
U-Net	54,940,997.0	2.027
Y-Net	99,389,447.0	3.667
TransforCNN	82,892,014.0	3.058
E-Net	80,858,216.0	2.983

## Data Availability

The image stacks corresponding to the XCT stack and the TransforCNN result are available at https://doi.org/10.6078/D1FM8J, and they are compressed to comply with storage footprint and public software for fast data visualization.

## Sample Availability

XCT image samples from the compounds are available from the authors.

## Abbreviations

The following abbreviations are used in this manuscript:

CNN	Convolutional Neural Network
E-Net	Ensemble Network
LMB	Lithium metal battery
mDSC	mean Dice Similarity Coefficient
mIoU	mean Intersection over Union
MOPS	Multi-Scale Oriented Patches
NLP	Natural Language Processing
SIFT	Scale-Invariant Feature Transform
TransforCNN	Transformer Convolutional Neural Network
XCT	X-ray Computed Tomography
